# Design, Synthesis, and Evaluation of Amphiphilic Cyclic and Linear Peptides Composed of Hydrophobic and Positively-Charged Amino Acids as Antibacterial Agents

**DOI:** 10.3390/molecules23102722

**Published:** 2018-10-22

**Authors:** Neda Riahifard, Saghar Mozaffari, Taibah Aldakhil, Francisco Nunez, Qamar Alshammari, Saud Alshammari, Jason Yamaki, Keykavous Parang, Rakesh Kumar Tiwari

**Affiliations:** 1 Center for Targeted Drug Delivery, Department of Biomedical and Pharmaceutical Sciences, Chapman University School of Pharmacy, Harry and Diane Rinker Health Science Campus, Irvine, CA 92618, USA; riahi103@mail.chapman.edu (N.R.); mozaf100@mail.chapman.edu (S.M.); aldak100@mail.chapman.edu (T.A.); nunez138@mail.chapman.edu (F.N.); qalshammari@chapman.edu (Q.A.); alshammari@chapman.edu (S.A.); 2 Department of Pharmacy Practice, Chapman University School of Pharmacy, Harry and Diane Rinker Health Science Campus, Irvine, CA 92618, USA; yamaki@chapman.edu

**Keywords:** amphiphilic cyclic peptide, cationic, *E. coli*, hydrophobicity, methicillin-resistant *Staphylococcus aureus*

## Abstract

Antimicrobial peptides (AMPs) contain amphipathic structures and are derived from natural resources. AMPs have been found to be effective in treating the infections caused by antibiotic-resistant bacteria (ARB), and thus, are potential lead compounds against ARB. AMPs’ physicochemical properties, such as cationic nature, amphiphilicity, and their size, will provide the opportunity to interact with membrane bilayers leading to damage and death of microorganisms. Herein, AMP analogs of [R_4_W_4_] were designed and synthesized by changing the hydrophobicity and cationic nature of the lead compound with other amino acids to provide insights into a structure-activity relationship against selected model Gram-negative and Gram-positive pathogens. Clinical resistant strains of methicillin-resistant *Staphylococcus aureus* (MRSA) and *Escherichia coli* (*E. coli*) were used in the studies. Our results provided information about the structural requirements for optimal activity of the [R_4_W_4_] template. When tryptophan was replaced with other hydrophobic amino acids, such as phenylalanine, tyrosine, alanine, leucine, and isoleucine, the antibacterial activities were significantly reduced with MIC values of >128 µg/mL. Furthermore, a change in stereochemistry caused by d-arginine, and use of *N*-methyltryptophan, resulted in a two-fold reduction of antibacterial activity. It was found that the presence of tryptophan is critical for antibacterial activity, and could not be substituted with other hydrophobic residues. The study also confirmed that cyclic peptides generally showed higher antibacterial activities when compared with the corresponding linear counterparts. Furthermore, by changing tryptophan numbers in the compound while maintaining a constant number of arginine, we determined the optimal number of tryptophan residues to be four, as shown when the number of tryptophan residues increased, a decrease in activity was observed.

## 1. Introduction

The rapid increase in the multidrug-resistant pathogens around the world has led to a pressing demand for the discovery of new generations of antibacterial compounds. The failure of the most potent antibacterial agents against superbugs, and the preview of future antibacterial treatments, emphasize the necessity of developing new antibacterial agents [1]. Peptides are potential therapeutic candidates with broad-spectrum activity against targeted organisms, ranging from viruses to parasites. Antimicrobial peptides (AMPs) are a class of synthetic and natural peptides with good activity and low toxicity [2]. Cationic amphipathic peptides are a subgroup of AMPs that have been studied consistently in recent decades [3]. Many natural AMPs have been found in both eukaryotes and prokaryotes. In mammals, they are generally present in the organs that are exposed to airborne pathogens. Thus, AMPs are considered as the first line of defense [4]. Cationic AMPs are a potent class of compounds, which have been reported as therapeutics agents against antibiotic-resistant pathogens. AMPs are important due to their mode of action as effectors, regulators of the immune system, and ability to inhibit bacterial cell growth [5]. Many studies support their effectiveness against resistant pathogens [6]. The evolution of pathogens that are resistant to AMPs has been attributed to fundamental changes in membrane composition, which is a slow process. The amphiphilicity, cationic nature, and hydrophobicity of AMPs are three essential features for antibacterial activity. Cationic AMPs have a secondary structure that contributes to their antibacterial activity. Furthermore, the position of charged amino acids and the size of the hydrophilic and hydrophobic regions can influence AMPs’ antibacterial activities.

**Figure 1:**
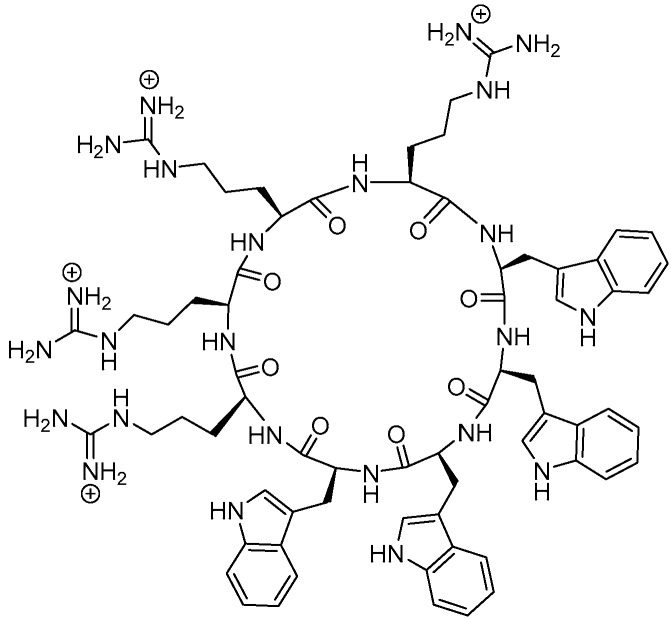
Chemical structures of amphiphilic cyclic peptide [R_4_W_4_].

Synthesis of AMPs by modifying the structures of amino acids is relatively easy [7]. Change in the structure of natural and synthetic AMPs can be used to develop new active compounds with optimal activity and stability. Developing small-sized linear or cyclic peptides for AMP application is more accessible than the complex AMPs isolated from natural products containing disulfide bonds [8]. The cyclic peptides are polypeptides chains containing a cyclic structure synthesized by linking the carboxylic acid and amino groups in the peptide. The cyclic peptides were more potent in comparison with the linear form due to their specific interactions with receptor surface [9]. Due to the constrained structure of the cyclic peptide, they have also been found to be more stable against proteases [10]. Moreover, some cyclic peptides have the ability to penetrate cells [11] or increase bacterial membrane permeability and change their membrane structure [12,13,14]. Hence, they could potentially be used as dual action treatments, i.e., as membrane permeability agents and antibiotics. This dual property was demonstrated by amphiphilic cyclic peptide [R_4_W_4_] (**7**) (Figure 1), which showed a MIC value of 2.67 µg/mL against MRSA (ATCC 43300) [15]. We have previously tested [RW]_4_, which has alternate tryptophan and arginine residues. However, it did not demonstrate any significant antibacterial activities. The amphiphilic nature of the peptide with hydrophobic groups on one side and the positively charged residues in the opposite side contribute significantly to the antibacterial activity. In this study, we developed a SAR based on the selection of appropriately-charged and hydrophobic amino acids, in order to improve the antibacterial profile of [R_4_W_4_] against selected Gram-negative and Gram-positive resistant bacteria.

We designed several series of peptides, in which we have rationally modified the sequence or stereochemistry of the compound, and then used standardized assays to evaluate antibacterial activity. We expected that peptides containing hydrophobic residues and charged residues in an appropriate sequence might have inherent cell-penetrating and antibacterial properties. To examine this hypothesis, we designed and synthesized cyclic and linear peptides composed of positively-charged arginine and different hydrophobic residues. The advantages of this strategy could be: (i) Arginine amino acids in the structure of the peptides could interact with negatively-charged parts in the cell membranes such as anionic phosphate residues, and cause cell rupture; (ii) The use of peptides to treat a bacterial infection could be safer because the peptides contain natural amino acids that can be biodegradable by proteolytic enzymes; (iii) The cyclic nature of the peptide makes it more stable to hydrolysis versus the corresponding linear peptides in the presence of exopeptidases, due to the lack of both amino and carboxyl termini.

Based on the reported data, the R_4_X_4_ sequence was selected as the model scaffold [15]. Tryptophan, leucine, isoleucine, alanine, phenylalanine, tyrosine, and *N*-methyl tryptophan were selected as hydrophobic amino acids in the sequence of the peptides. Besides changes in the hydrophobic domain of the lead compound, arginine was replaced with glutamic acid and lysine to study the importance of the positive charge. Furthermore, d-arginine was used instead of l-arginine to evaluate the molecular configurational change impact on antibacterial activity.

Fmoc solid-phase synthesis was applied to accomplish the synthesis of all the proposed peptides. The goal was to generate several compounds for comparisons with [R_4_W_4_] in order to determine the critical residues in the peptide sequence for antibacterial activity. Square brackets [ ] and parentheses ( ) were used for the cyclic and linear peptides, respectively.

## 2. Results and Discussion

### 2.1. Chemistry

**Figure 2:**
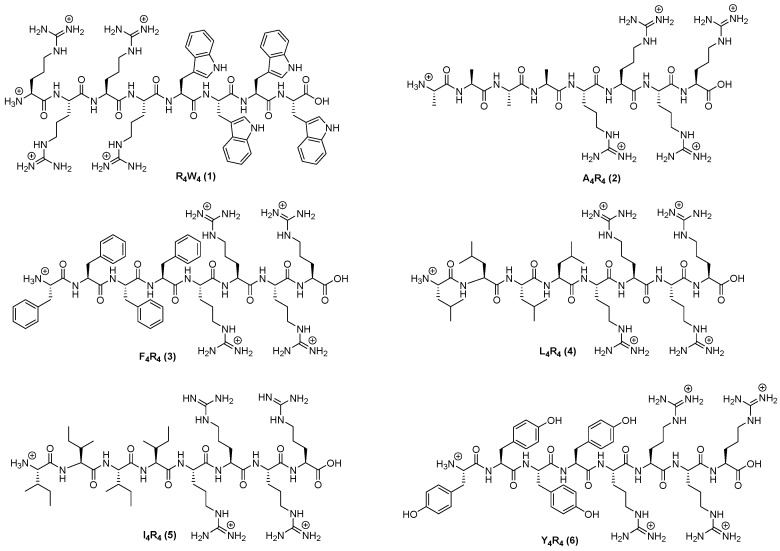
Chemical structures of linear peptides X_4_R_4_.

**Figure 3:**
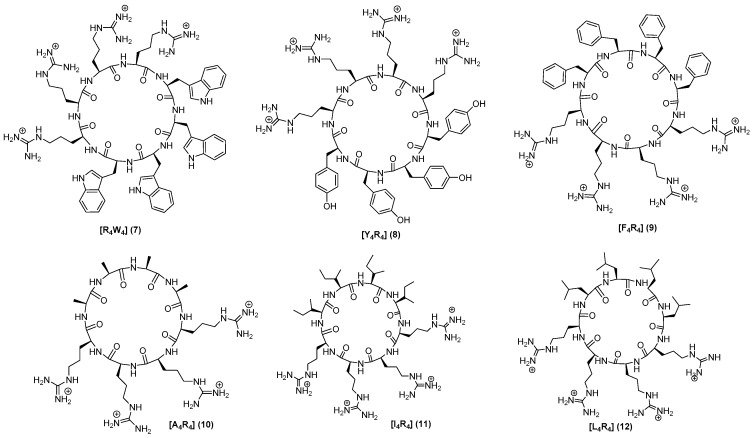

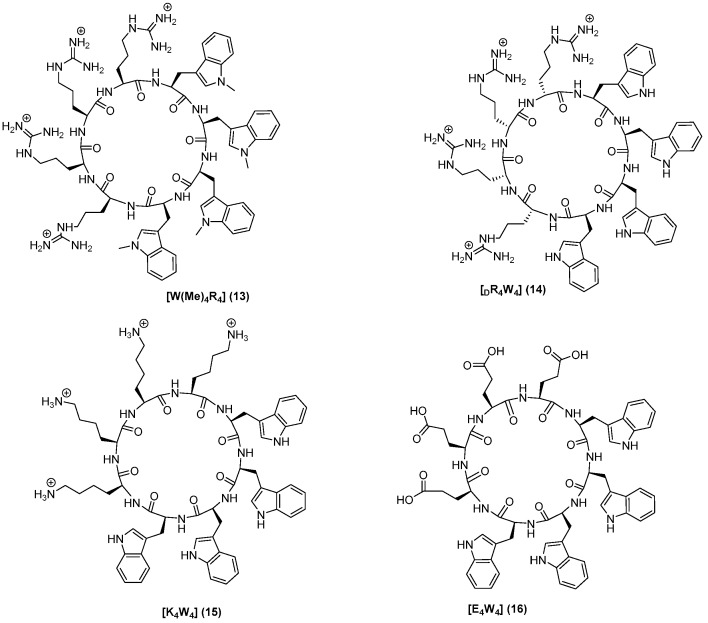
Chemical structures of cyclic peptides.

**Figure 4:**
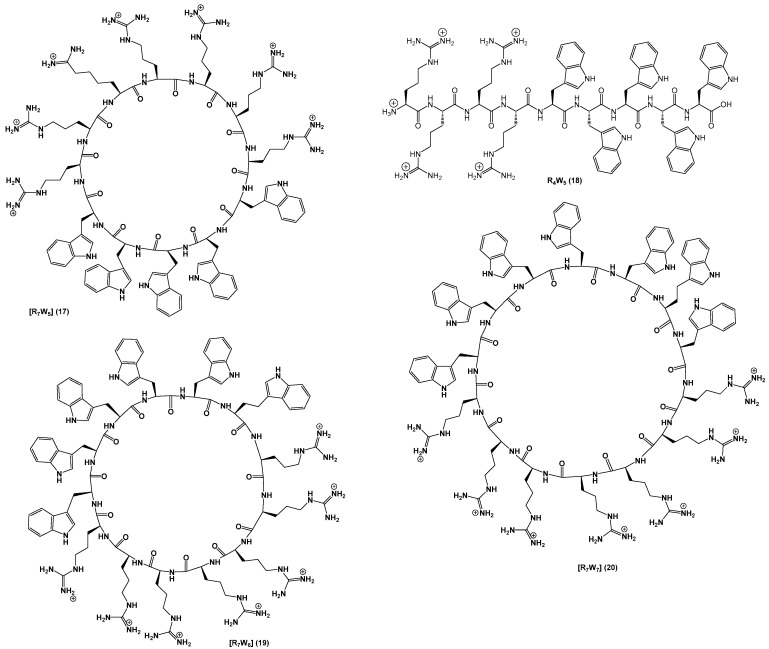
Chemical structures of [R_7_W_5_], R_4_W_5_, [R_7_W_6_], and [R_7_W_7_].

All 20 peptides were designed to establish SAR and were synthesized using Fmoc/tBu solid-phase peptide synthesis. Peptides (**1**–**14)** were designed to determine the impact of any changes in hydrophobic moieties. Peptide [E_4_W_4_] (**15**) was designed to have hydrophobic and negatively-charged residues. Peptide [K_4_W_4_] (**16**) had a cyclic structure containing tryptophan and lysine as a positive charge for comparative studies. Peptides [R_4_W(Me)_4_] (**13**) and [(_d_R)_4_W_4_] (**14**) containing *N*-methyl tryptophan and d-arginine, respectively, were synthesized to determine the effect of *N*-methylation and changes on stereochemistry in tryptophan. Furthermore, three more peptides (**19**–**21**) with a different number of tryptophan were synthesized. The chemical structures of all synthesized linear and cyclic peptides are depicted in Figure 2, Figure 3 and Figure 4.

**Scheme 1:**
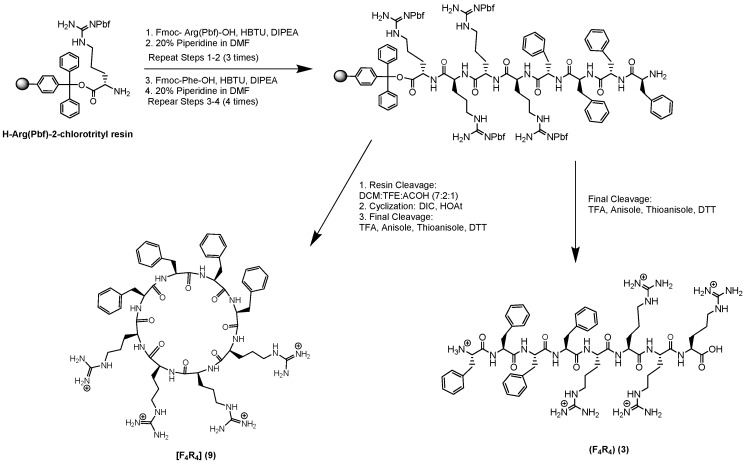
Synthesis of linear (R_4_F_4_) (**3**) and cyclic [R_4_F_4_] (**9**) as representative examples.

Scheme 1 depicts the synthesis of linear (R_4_F_4_) (**3**) and cyclic [R_4_F_4_] (**9**) peptides as representative examples. The linear peptide sequence was first assembled on the solid phase. The cleavage from the resin was conducted using a cleavage cocktail of dichloromethane (DCM)/2,2,2-trifluoroethanol (TFE)/acetic acid [7:2:1 (*v*/*v*/*v*)]. The cyclization was carried out in the presence of HOAT and DIC in an anhydrous DMF/DCM [4:1 (*v*/*v*)]. After final cleavage with the cleavage cocktail reagent R using trifluoroacetic acid (TFA)/thioanisole/1,2-ethanedithiol (EDT)/anisole cocktail [90:5:3:2 (*v*/*v*/*v*/*v*)], the peptides were precipitated and purified using reversed-phase high-performance liquid chromatography (RP-HPLC). The structure of peptides was confirmed using MALDI-TOF spectrometer (see Supplementary Materials). The chemical structures and sequences of all the synthesized peptides are shown in Figure 2, Figure 3 and Figure 4. All the final compounds were >95% pure, as shown by HPLC.

### 2.2. Antibacterial Assay

To evaluate the antibacterial activity of all the synthesized peptides, the minimum inhibitory concentration (MIC) was determined using the micro broth dilution method (Table 1), as per the Clinical Laboratory Standards Institute (CLSI). The MIC is a quantitative measurement of the minimum concentration of a compound required to inhibit visible bacterial growth. MIC values for all 20 peptides were measured against *E. coli* (ATCC 25922) and MRSA (Los Angeles County clone (LAC)) (Table 1). These two bacterial strains represent Gram-negative and Gram-positive pathogens. MRSA is one of the most challenging Gram-positive bacteria that leads to a life-threatening infection, while *E. coli* is a Gram-negative bacteria that has the ability to use gene mutations and multidrug efflux pumps that can result in multidrug resistance [16].

The evaluation of antibacterial activity showed that tryptophan is required for antibacterial activity, as replacing tryptophan with other amino acids demolishes the activity. All the compounds containing arginine and substituted tryptophan with other hydrophilic and hydrophobic amino acids in the cyclic peptides [R_4_X_4_] and linear peptides X_4_R_4_, (where X is tyrosine, leucine, isoleucine, alanine, phenylalanine) **(1**–**12)** had very high MIC values, none close MIC to [R_4_W_4_]. We concluded that the presence of tryptophan is vital for antibacterial activity in the peptide. Moreover, the evaluation of antibacterial activities also revealed that increasing the number of tryptophan and arginine in the compound improved the activity, since the MIC value for [R_7_W_5_] was 32 μg/mL, for [R_7_W_6_] was 16 μg/mL, and for [R_7_W_7_] was 8 μg/mL, whereas [R_4_W_4_] still showed higher activity 4 μg/mL for MRSA. Thus, we concluded that R_4_ is more optimal in comparison with seven arginine residues (R_7_). Furthermore, arginine was replaced with d-arginine, to study stereochemistry of the compound and its impact on antibacterial activity. Comparison of the MIC value of [_d_R_4_W_4_] containing d-arginine with lead compound [R_4_W_4_] demonstrated that this molecular configuration reduced the activity by two-fold. These data indicate that R_4_, arginine with L configuration, and unmodified tryptophan, are required for generating optimal activity. Replacing arginine with glutamic acid in [E_4_W_4_] abolished the activity completely, indicating that positively-charged residues are required for antibacterial activity. Replacement of arginine to lysine in [K_4_W_4_] reduced the activity by two-fold. Further structure modifications are required to optimize the antibacterial activity of [R_4_W_4_]. In general, the peptides were significantly more potent against MRSA versus *E. coli*.

### 2.3. Cytotoxicity Assay

**Figure 5:**
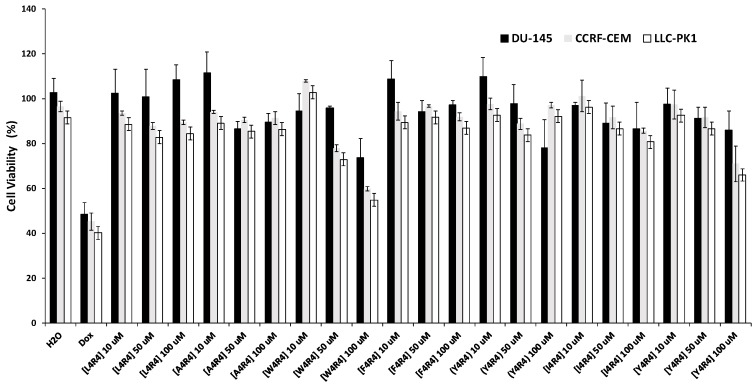
Cytotoxicity of peptides against three cell lines (prostate cancer DU-145, human leukemia CCRF-CEM, and kidney cell line LLC-PK1) using the MTS assay after 24 h incubation.

The cytotoxicity of a number of the synthesized peptides was determined using the MTS proliferation assay. Human prostate cancer (DU-145), human leukemia (CCRF-CEM), and normal kidney (LLC-PK1) cells were used as cell lines. Cytotoxicity assays were conducted at three concentrations (10, 50, and 100 µM). At a concentration of 10 μM, the compounds exhibited more than 84% cell viability in both normal and cancer cell lines (Figure 5), and were not significantly cytotoxic. The peptide treatment affected CCRF-CEM cells more than LLC-PK-1. [R_4_W_4_] demonstrated toxicity at higher concentrations (100 µM), and reduced the cell viability to 54.83%, 59.83%, and 73.48% for LLC-PK1, CCR-CEM, and DU-145 cell lines, respectively. However, this concentration is much higher than that used for the antibacterial assay, suggesting that the compound is more selective toward tested bacterial cells than mammalian cells. Compound **7** had a MIC value of 4 µM against MRSA, that is much lower than the cytotoxic concentration. Other peptides did not show significantly high toxicity at a higher concentration, which is consistent with their absence of antibacterial activity.

## 3. Materials and Methods

### 3.1. Materials

Protected l-amino acids and amino acids preloaded on 2-chlorotrityl resin were purchased from AAPPtec LLC, Louisville, KY, USA. Other required materials, such as trifluoroacetic acid (TFA), dimethylformamide (DMF), piperidine, DIPEA, and HPLC grade solvents for peptide purification such as methanol and acetonitrile were purchased from Sigma-Aldrich Co (Milwaukee, WI, USA). High-resolution MALDI-TOF (GT 0264) from Bruker Daltonics, Inc., San Jose, CA, USA was used to confirm the chemical structures of final products. Reversed-phase HPLC (Shimadzu Scientific Instruments, Inc, Pleasanton, CA, USA (LC-20AP)) was used to purify the synthesized compounds. A gradient system of water and acetonitrile containing 0.1% TFA and a reversed-phase preparative column (XBridge BEH130 Prep C18) with the flow rate of 10 mL/min was used in the HPLC. The bacterial strain Methicillin-Resistant *Staphylococcus aureus* (Los Angeles County (LAC) clone) and *E. Coli* (ATCC 25922) were acquired from the Los Angeles Public Health Department (Los Angeles, CA, USA) and American Type Culture Collection (ATCC), respectively. All the media for bacterial experiments were obtained from Hardy Diagnostics (Santa Maria, CA, USA).

### 3.2. Synthesis of Peptides

All the peptides were synthesized using our previously-reported synthesis [15,17]. The peptides were synthesized via manual method by applying a Chemglass peptide synthesis vessel (#CG1860) and bubbling anhydrous nitrogen gas. Synthesis of linear (F_4_R_4_) (**3**) and cyclic [F_4_R_4_] (**9**) are explained here as representative examples. All other peptides were synthesized using a similar procedure. The preloaded amino acid on resin, H-Arg(Pbf)-2-chlorotrityl resin (0.44 meq/g, 0.3 mmole, 681 mg) was used as solid support containing the first amino acid in the reaction vessel, and was swelled using DMF (50 mL, 30 min × 2) with bubbling nitrogen gas. The solvent was drained off, and Fmoc-Arg(Pbf)-OH (1.2 mmol, 778 mg) was added for the coupling using a coupling and activating reagents HBTU (1.2 mmol, 455 mg), DIPEA (2.4 mmol, 1.0 mL), respectively, in DMF (15 mL) for 2 h. The resin was subsequently washed with DMF three times. (25 mL × 3). Deprotection of the Fmoc group was conducted by using 20% piperidine in DMF (*v*/*v*) two times (20 mL, 10 min × 2). The resin was washed three times with DMF (25 mL × 3). The couplings were performed two more times using Fmoc-Arg(Pbf)-OH as described above. Four additional couplings were carried out in the presence of Fmoc-Phe-OH (1.2 mmol, 465 mg), DIPEA (2.4 mmol, 1.0 mL), HBTU (1.2 mmol, 455 mg) in DMF (15 mL) for 2 h. Finally, the *N*-terminal Fmoc protecting group was deprotected in the presence of piperidine. The peptide-attached resin was subsequently washed with DCM (25 mL × 3). The resin was divided into two parts. The first part was washed by MeOH (15 mL × 3) and used for cleavage of the fully-deprotected linear peptide by shaking and mixing with cleavage cocktail reagent R for 2 h at room temperature. Reagent R contains trifluoroacetic acid/anisole/1,2-ethanedithiol/thioanisole cocktail (90:2:3:5 (*v*/*v*/*v*/*v*), 10 mL). Subsequent centrifugation and precipitation with cold ether were used to obtain the crude linear peptide. The second part of the peptidyl resin was used to synthesize cyclic peptide. The protected peptide on the resin was cleaved from the resin with agitation of peptidyl resin with a cleavage cocktail that kept the side chain protecting groups intact. A mixture of trifluoroethanol, acetic acid, and dichloromethane, (TFE:AcOH:DCM; 1:2:7, *v*/*v*/*v*, 50 mL) was added to the resin and the mixture was shaken for 3 h. After evaporation of the filtrate, DCM (2 × 15 mL) and hexane (2 × 20 mL) were added to the residue to make sure that the acetic acid was removed from the mixture successfully. The crude product was precipitated as a white solid. After drying under vacuum overnight, HOAt (0.6 mmol, 4 equiv, 68 mg), anhydrous DMF (200 mL) and anhydrous DCM (50 mL) were added. The mixture was stirred, and DIC (0.66 mmol, 4.4 equiv, 103.5 µL) was added dropwise to the mixture to carry out the cyclization. The stirring was continued under nitrogen overnight. MALDI analysis was used to confirm the completion of the cyclization. After removal of the solvents under reduced pressure, complete deprotection of the side chains was conducted by agitation in the presence of cleavage cocktail R, as used for linear peptide followed by precipitation of crude cyclic peptide. The crude peptides (linear and cyclic) were purified by RP-HPLC. A gradient system from 0 to 100% acetonitrile (CH_3_CN) and water containing 0.1% (*v*/*v*) TFA was used for the purification. The elution time of 1 h, a wavelength of 218 nm, and a flow rate of 10.0 mL/min, were used for the HPLC. The molecular masses of all the synthesized peptides are as follow:

R_4_W_4_ (**1**) MALDI-TOF (*m*/*z*): C_68_H_90_N_24_O_9_: calcd, 1386.7323; found, 1391.1422 [M + 5H]^+^; A_4_R_4_ (**2**) MALDI-TOF (*m*/*z*): C_36_H_70_N_20_O _9_: calcd, 926.5635; found, 927.4231 [M + H]^+^; (F_4_R_4_) (**3**) MALDI-TOF (*m*/*z*):C_60_H_86_N_20_O_9_: calcd, 1230.6887; found, 1231.4021 [M + H]^+^; L_4_R_4_ (**4**) MALDI-TOF (*m*/*z*): C_48_H_94_N_20_O_9_: calcd, 1094.7513; found, 1095.6045 [M + H]^+^; I_4_R_4_ (**5**) MALDI-TOF (*m*/*z*): C_48_H_94_N_20_O_9_: calcd, 1094.7513; found, 1095.7431 [M + H]^+^; Y_4_R_4_ (**6**) MALDI-TOF (*m*/*z*): C_60_H_86_N_20_O_13_: calcd, 1294.6683; found, 1295.2881 [M + H]^+^; [W_4_R_4_] (**7**) MALDI-TOF (*m*/*z*): C_68_H_88_N_24_O_8_: calcd, 1368.7217; found, 1369.4419 [M + H]^+^; [R_4_Y_4_] (**8**) MALDI-TOF (*m*/*z*): C_60_H_84_N_20_O_12_: calcd, 1276.6578; found, 1277.3021 [M + H]^+^; [F_4_R_4_] (**9**) MALDI-TOF (*m*/*z*):C_60_H_84_N_20_O_8_: calcd, 1212.6781; found, 1213.3169 [M + H]^+^; [A_4_R_4_] (**10**) MALDI-TOF (*m*/*z*): C_36_H_68_N_20_O_8_: calcd, 908.5529; found, 908.3209 [M]^+^; [I_4_R_4_] (**11**) MALDI-TOF (*m*/*z*): C_48_H_92_N_20_O_8_: calcd, 1076.7407; found, 1077.5388 [M + H]^+^; [L_4_R_4_] (**12**) MALDI-TOF (*m*/*z*): C_48_H_92_N_20_O_8_: calcd, 1076.7407; found, 1077.5388 [M + H]^+^; [W_(Me)4_R_4_] (**13**) MALDI-TOF (*m*/*z*): C_72_H_96_N_24_O_7_: calcd, 1424.7843; found, 1426.0493 [M + 2H]^+^; [_D_R_4_W_4_] (**14**) MALDI-TOF (*m*/*z*): C_68_H_88_N_24_O_8_: calcd, 1368.7217; found, 1369.4419 [M + H]^+^; [K_4_W_4_] (**15**) MALDI-TOF (*m*/*z*): C_68_H_88_N_16_O_8_: calcd, 1256.6971; found, 1257.7218 [M + H]^+^; [W_4_E_4_] (**16**) MALDI-TOF (*m*/*z*): C_64_H_68_N_12_O_16_: calcd, 1260.4876; found, 1261.9298 [M + H]^+^; [R_7_W_5_] (**17**) MALDI-TOF (*m*/*z*): C_97_H_134_N_38_O_12_: calcd, 2023.1043; found, 2024.5960 [M + H]^+^; R_4_W_5_ (**18**) MALDI-TOF (*m*/*z*): C _79_H_100_N_26_O_10_: calcd, 1572.8116; found, 1577.3938 [M + 5H]^+^; [R_7_W_6_] (1**9**) MALDI-TOF (*m*/*z*): C_108_H_144_N_40_O_13_: calcd, 2209.1837; found, 2212.0489 [M + 2H]^+^; [R_7_W_7_] (**20**) MALDI-TOF (*m*/*z*): C_119_H_154_N_42_O_14_: calcd, 2395.2630; found, 2397.9160 [M + 2H]^+^.

### 3.3. Antibacterial Assay

Two bacterial strains, methicillin-resistant *Staphylococcus aureus* (LAC) and *Escherichia coli* (ATCC 25922) were used for the evaluation of the antibacterial activities of the peptides. The activities were compared to control antibiotics (vancomycin and meropenem) and [R_4_W_4_] using our previously-published protocol [17]. MRSA and *E. coli* were inoculated into Mueller Hinton (MH) broth (5 mL) at 37 °C. After shaking in an orbital shaker (175 rpm) overnight, the cultured suspension was diluted using normal saline (5 mL). Dilution was continued until turbidity of 0.5 McFarland (1.5 × 10^8^ bacterial cell CFU/ml) was achieved. An amount of 40 μL of the McFarland solution was then added to 5980 μL of MH media to afford 1/150 dilution. The majority of peptides were then dissolved in distilled water to generate 256 μg/mL solutions. However, some of the peptides were dissolved in NH_4_HCO_3_ (5 mM) to increase the solubility. Minimal inhibitory concentrations (MIC) values were obtained using the broth micro dilution assay. In brief, first, the peptides and controls (200 μL) were added to the first column of the 96 well plate. Second, 2-fold serial dilutions of the compound were accomplished by adding media (100 μL) to other wells. After the serial dilution, the bacterial solution (100 μL) was added to all the wells. Finally, the microtiter plates were incubated statically at 37 °C overnight. MIC values were obtained by observing the minimal concentration at which bacterial growth was not visible. All the determinations were carried out in triplicate.

### 3.4. Cytotoxicity Assay of Peptides

The cytotoxicity of peptides was evaluated against human leukemia cancer cells (CCRF, ATCC No. CCL-119), human prostate cancer cells (DU145, ATCC No. HTB-81), and a human normal kidney (LLC-PK1, ATCC No. CRL-1392) cell line by MTS assay using Cell Titer and 96 assay plate. All the cells were cultured as per the manufacturer’s guidelines. After cell cultures, the plating of the cells in 96 well plates was carried out at 37 °C overnight with a density of 5000 cells per well in 0.1 mL of growth medium. Different concentrations of peptides were incubated with the cells for 24 h in a humidified atmosphere of 5% CO_2_. The cells without compounds were used as experimental controls. The cytotoxicity of the peptides was compared with those of standard drug antibiotics. MTS was added after incubation, and incubation was continued for 2 h. The absorbance of the Formazan product at 490 nm was determined using a microplate reader. Cytotoxicity percentage was determined as (Optical density (OD) value of untreated cells − OD value of treated cells)/OD value of untreated cells = 100%.

## 4. Conclusions

Based on the evaluation of the antibacterial activity of a newly-synthesized library of 20 peptides, we concluded that [R_4_W_4_] is still the most active antibacterial peptide, with a MIC value of 4 μg/mL. In general, the cyclic peptides showed higher antibacterial activities when compared with their corresponding linear counterparts. The evaluation of antibacterial activity revealed improved activity with the number of tryptophan residues increases in [R_7_W_5_], [R_7_W_6_] and [R_7_W_7_]. [R_4_W_4_] still showed higher activity, suggesting that the number of arginine is optimal in the structure. The data indicate that R_4_, arginine with L-configuration, and unmodified tryptophan were required to generate optimal activity. Further structure modification is required to optimize the antibacterial activity of [R_4_W_4_]. This work provides insights into developing cyclic peptides as antibacterial agents, and the importance of specific hydrophobic and positively charged residues in generating optimal antibacterial activity.

## Figures and Tables

**Table 1:** Antibacterial assay against Gram-negative and Gram-positive bacteria.

Compounds	MIC (μg/mL) ^#^ MRSA (LAC)	MIC (μg/mL) ^#^ *E. coli* (ATCC 25922)
R_4_W_4_ (1)	32	64
A_4_R_4_ (2)	>128	>128
F_4_R_4_ (3)	>128	>128
L_4_R_4_ (4)	>128	>128
I_4_R_4_ (5)	>128	>128
Y_4_R_4_ (6)	>128	>128
[R_4_W_4_] (7)	4	16
[Y_4_R_4_] (8)	>128	>128
[F_4_R_4_] (9)	>128	>128
[A_4_R_4_] (10)	>128	>128
[I_4_R_4_] (11)	>128	>128
[L_4_R_4_] (12)	>128	>128
[W_(Me)4_R_4_] (13)	8	16
[_D_R_4_W_4_] (14)	8	16
[K_4_W_4_] (15)	8	16
[E_4_W_4_] (16)	>128	>128
[R_7_W_5_] (17)	32	64
R_4_W_5_ (18)_5_	16	32
[R_7_W_6_] (19)	16	64
[R_7_W_7_] (20)	8	32
Vancomycin Meropenem	1 ND	ND 0.25

^#^ MIC measurement was done in triplicate; ND = not determined.

## Supplementary Materials

The MALDI spectra of selected compounds are provided.
