# Supplementary material for: Design, Synthesis, and Evaluation of Amphiphilic Cyclic and Linear Peptides Composed of Hydrophobic and Positively-Charged Amino Acids as Antibacterial Agents
Source: Molecules. 2018 Oct 22;23(10):2722. doi: 10.3390/molecules23102722 (PMC6222377; doi:10.3390/molecules23102722)
Supplement: Supplementary file 1 [file molecules-23-02722-s001.pdf]

# Supplementary information

# Design, Synthesis, and Evaluation of Amphiphilic Cyclic and Linear Peptides Composed of Hydrophobic and Positively Charged Amino Acids As Antibacterial Agents

**Neda Riahifard [1], Saghar Mozaffari,[1] Taibah Aldakhil, [1] Francisco Nunez,[1] Qamar Alshmmari,[1] Saud Alshammari,[1] Jason Yamaki [2], Keykavous Parang [1,*] and Rakesh Kumar Tiwari [1,*]**

[1] Center for Targeted Drug Delivery, Department of Biomedical and Pharmaceutical Sciences, Chapman University School of Pharmacy, Harry and Diane Rinker Health Science Campus, Irvine, CA 92618, USA; riahi103@mail.chapman.edu (N.R.); mozaf100@mail.chapman.edu; aldak100@mail.chapman.edu; nunez138@mail.chapman.edu; qalshammari@chapman.edu; alshammari@chapman.edu

[2] Department of Pharmacy Practice, Chapman University School of Pharmacy, Harry and Diane Rinker Health Science Campus, Irvine, CA 92618, USA; yamaki@chapman.edu

* Correspondence: parang@chapman.edu (K.P.); tiwari@chapman.edu (R.T.); Tel.: +1-714-516-5483 (R.T.); +1-714-516-5489 (K.P.)

**Mass spectroscopy data of selected synthesized compounds:**

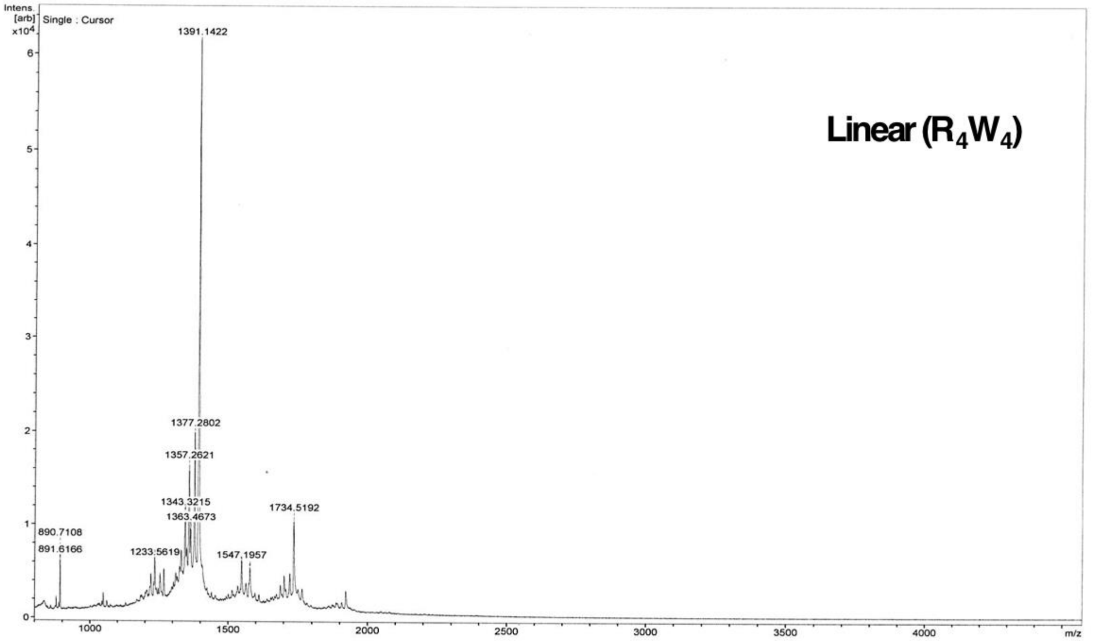


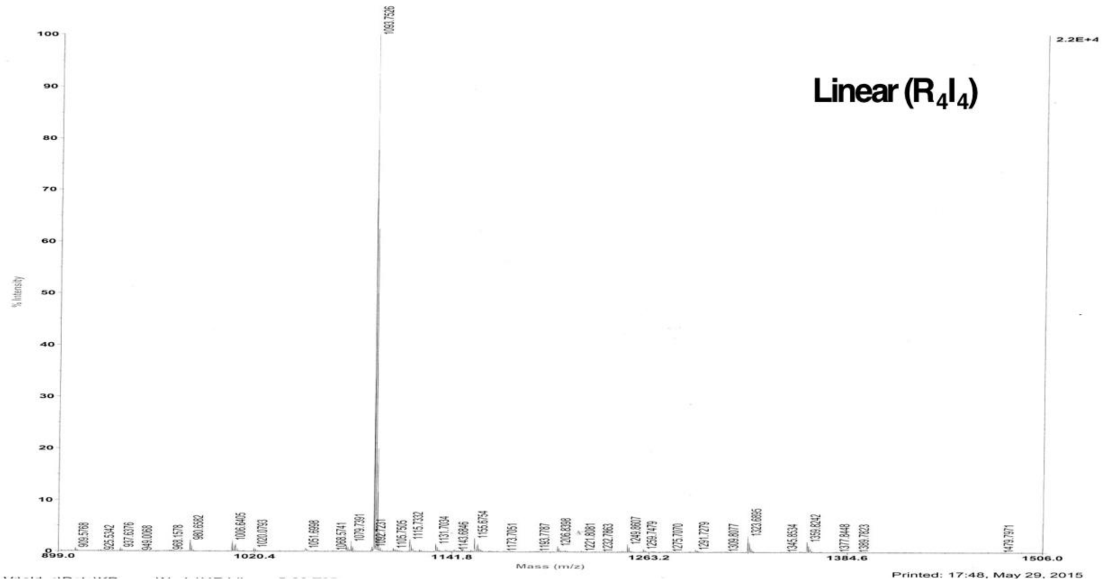

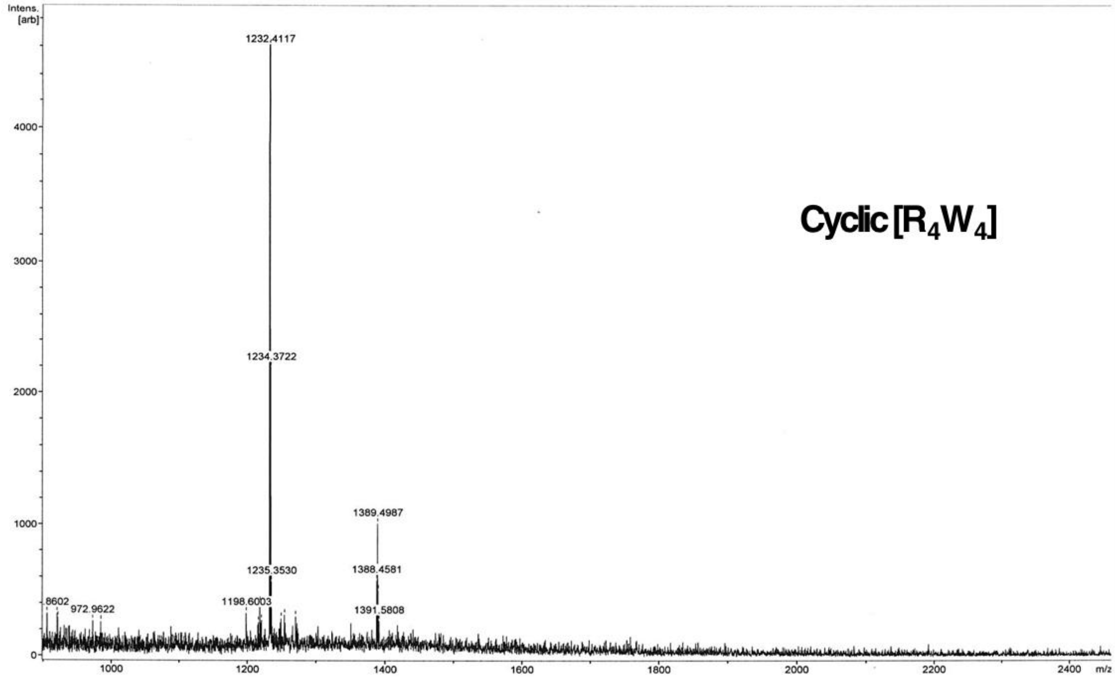
Intens. [arb]
1232.4117
1234.3722
1235.3530
1389.4987
1388.4581
1391.5808
1198.6003
972.9622
.8602
Cyclic [R4W4]
m/z

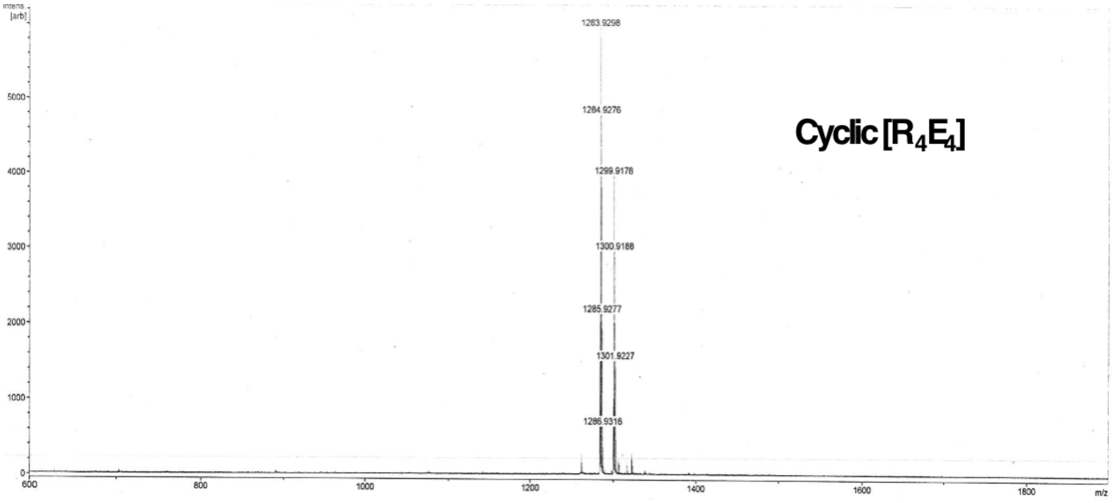
Intens. [arb]
1263.9298
1264.9276
1299.9176
1300.9188
1265.9277
1301.9227
1266.9316
Cyclic [R4E4]
m/z

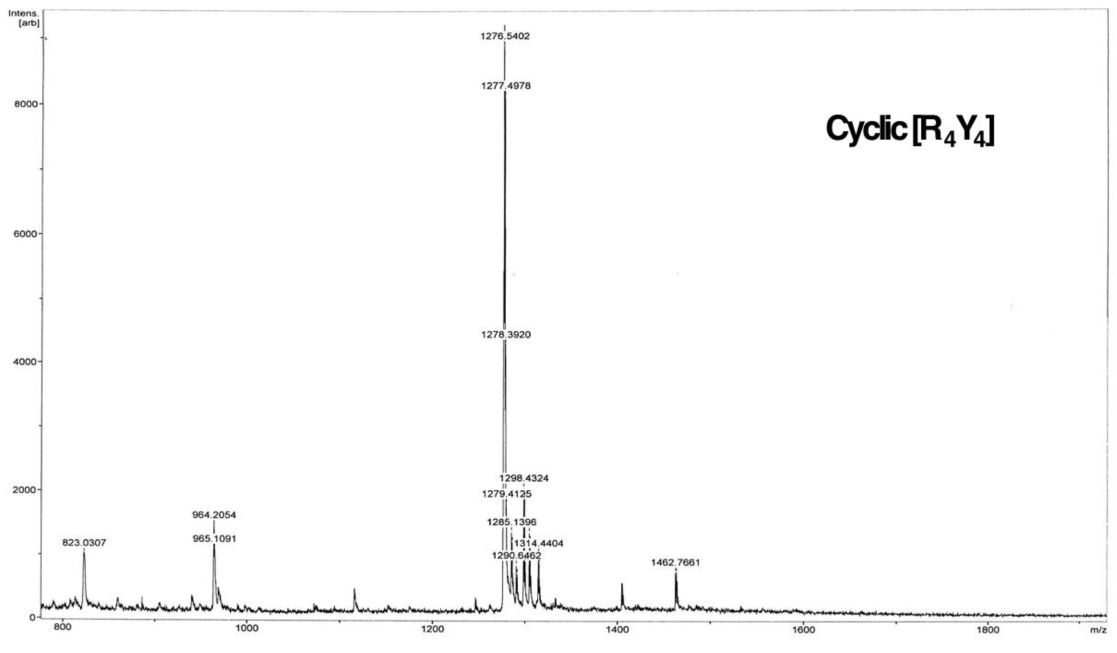
Intens. [arb]
Cyclic [R4Y4]
1276.5402
1277.4978
1278.3920
1298.4324
1279.4125
1285.1396
1314.4404
1290.6462
964.2054
965.1091
823.0307
1462.7661
m/z

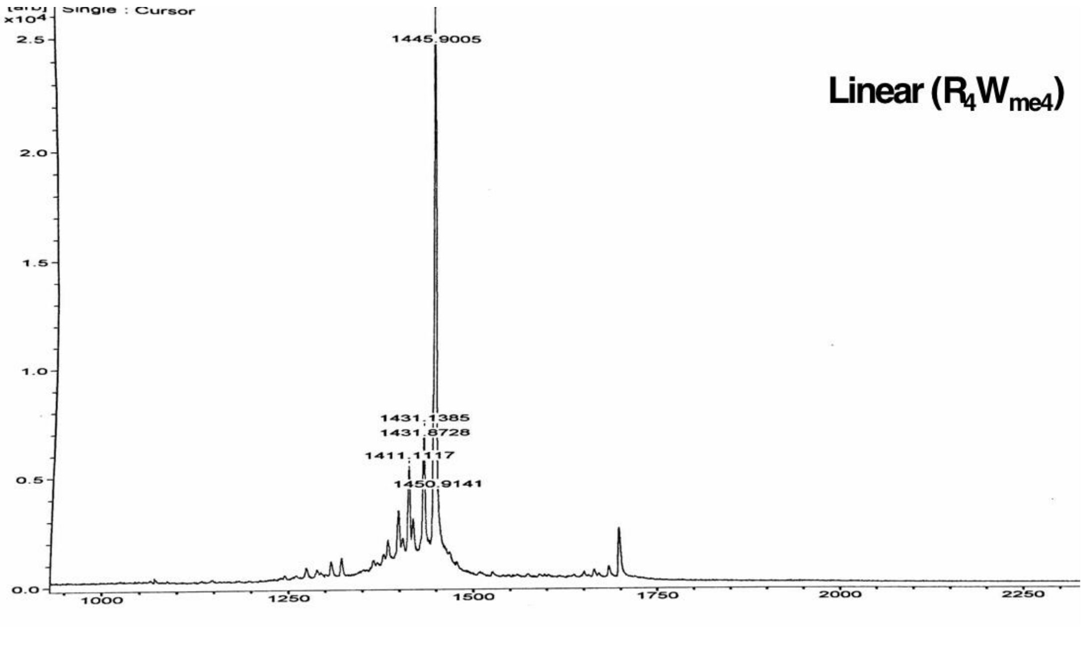
Single : Cursor
x10^4
Linear (R4Wme4)
1445.9005
1431.1385
1431.8728
1411.1117
1450.9141

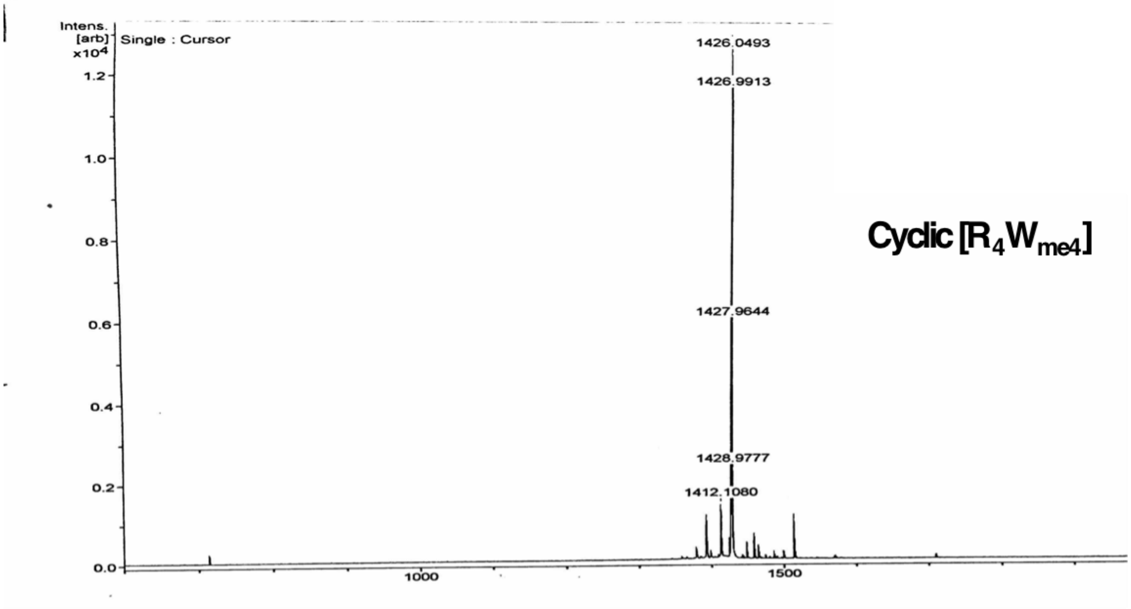
Intens.
[arb]
x10^4
Single : Cursor
1426.0493
1426.9913
1427.9644
1428.9777
1412.1080
1.2
1.0
0.8
0.6
0.4
0.2
0.0
1000
1500
Cyclic [R4Wme4]

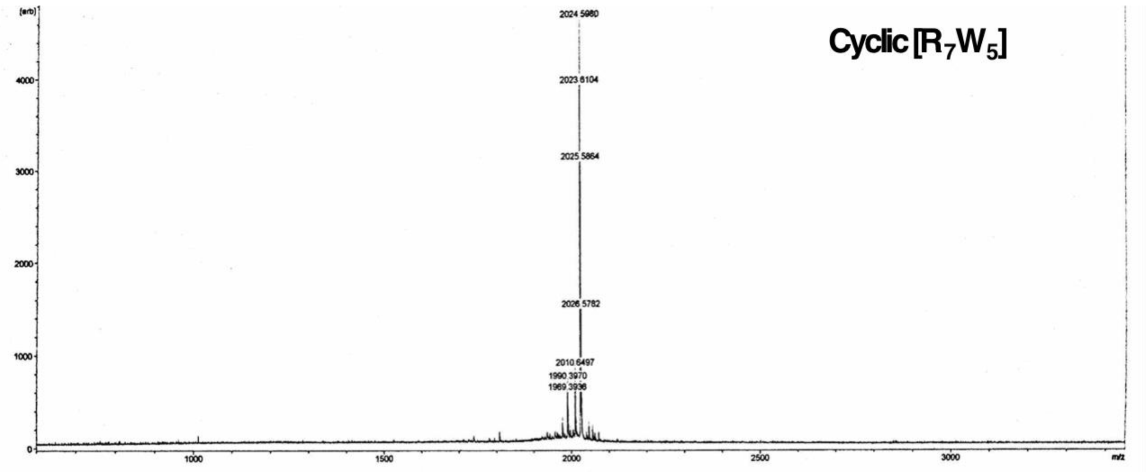
[arb]
Cyclic [R7W5]
2024.5980
2023.6104
2025.5864
2026.5782
2010.6497
1990.3970
1969.3936
4000
3000
2000
1000
0
1000
1500
2000
2500
3000
m/z

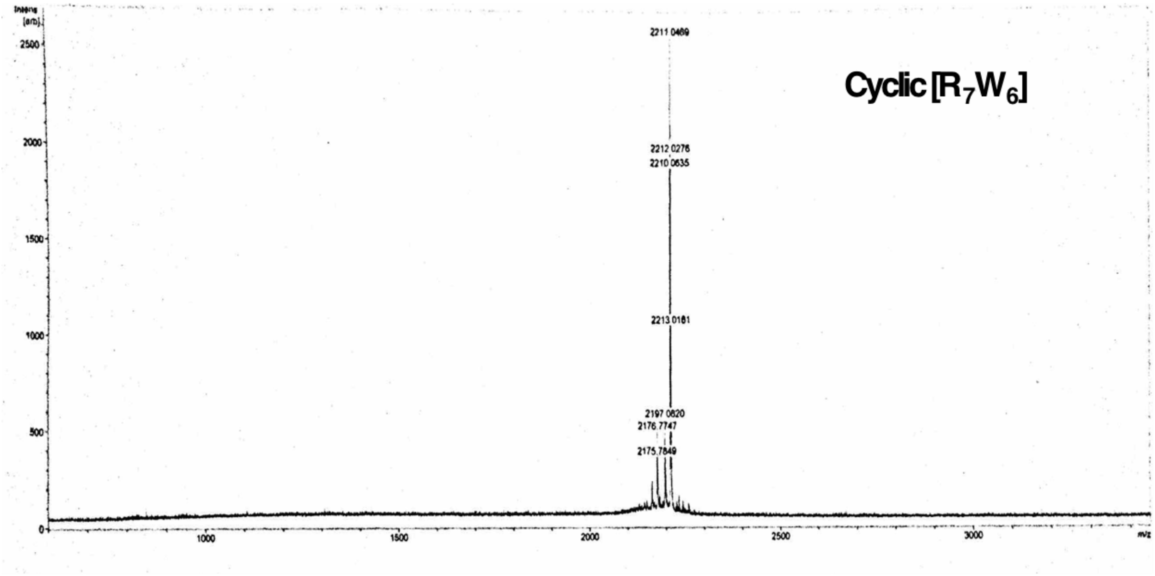
Intens. [arb.]
2211.0469
Cyclic [R7W6]
2212.0276
2210.0635
2213.0181
2197.0820
2176.7747
2175.7849
m/z

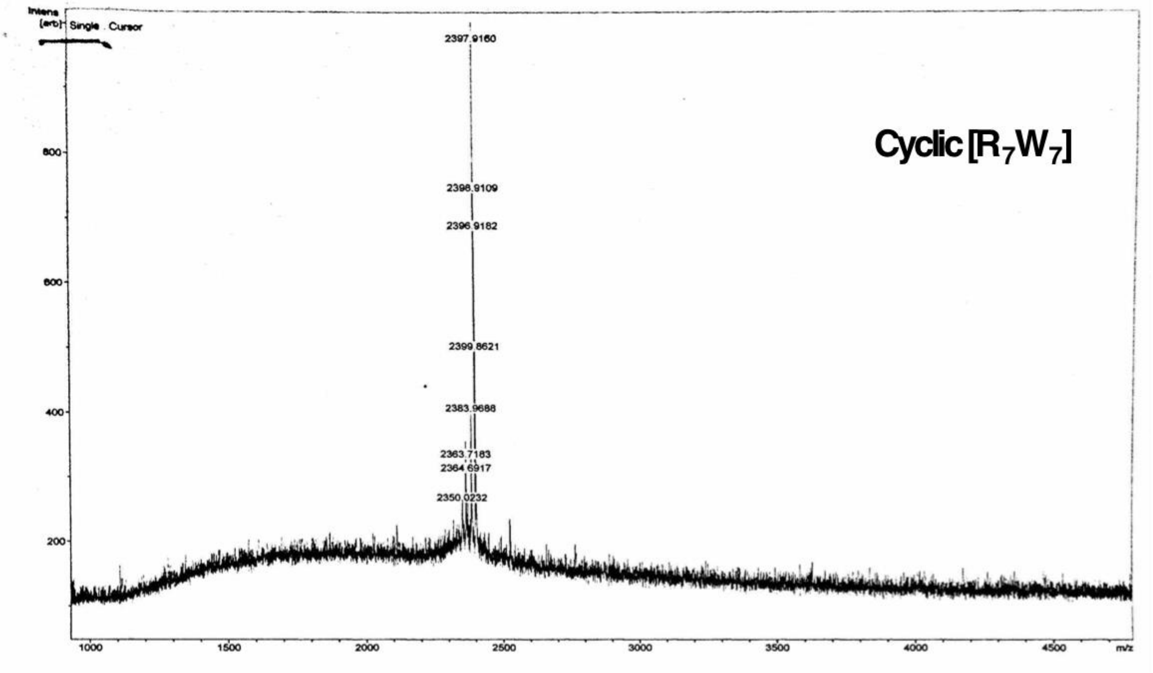
Intens. [arb.]
Single . Cursor
2397.9160
Cyclic [R7W7]
2398.9109
2396.9182
2399.8621
2383.9688
2363.7163
2364.6917
2350.0232
m/z
